# Diagnostic potential of routine brain MRI and high-resolution, multi-contrast vessel wall imaging in the detection of internal carotid artery dissection

**DOI:** 10.3389/fneur.2023.1165453

**Published:** 2023-05-11

**Authors:** Shanshan Xie, Yuncai Ran, Xiao Wang, Yong Zhang, Qichang Fu, Yanan Ren, Juanfang Liu, Zhongzhao Teng, Jingliang Cheng

**Affiliations:** ^1^Department of MRI, The First Affiliated Hospital of Zhengzhou University, Zhengzhou, China; ^2^Department of Intervention, The First Affiliated Hospital of Zhengzhou University, Zhengzhou, China; ^3^Department of Radiology, University of Cambridge, Cambridge, United Kingdom

**Keywords:** internal carotid artery, dissection, brain, magnetic resonance imaging, high-resolution vessel wall imaging, stroke

## Abstract

**Objective:**

Cervical artery dissection (CAD) is one of the major causes of stroke and most commonly occurs at the site of the extracranial internal carotid artery (ICA). This study aimed to assess the value of routine brain MRI, clinical information, and high-resolution, multi-contrast vessel wall MR imaging (hrVWI) for the timely detection of ICA dissection.

**Methods:**

A total of 105 patients with CAD and 105 without CAD were recruited for this study. The lesion type in the patients was determined based on images from different modalities, including brain MRI, magnetic resonance angiography (MRA), computed tomography angiography (CTA), digital subtraction angiography (DSA), ultrasonography, and hrVWI and clinical information. Each lesion was reviewed to determine the type following a stepwise procedure by referring to (1) brain MRI only; (2) brain MRI and clinical information; (3) hrVWI only; and (4) hrVWI, CTA, DSA, and clinical information.

**Results:**

Typical clinical presentations of patients with potential CAD include headache, neck pain, and/or Horner's syndrome. Representative imaging signs in the brain MRI included a crescentic or circular iso- or hyperintensity around the lumen, a curvilinear and isointense line crossing the lumen, or aneurysmal vessel dilation. Based on brain MRI alone, 54.3% (57/105) of the patients with CAD were correctly classified, and the accuracy increased to 73.3% (77/105) when clinical information was combined (*P* < 0.001) with high specificity and low sensitivity. Further analysis showed that hrVWI had the superior capability in detecting CAD, with a sensitivity and a specificity of 95.1% and 97.0%, respectively.

**Conclusion:**

The combination of brain MRI and clinical information could be used for the diagnosis of CAD; however, hrVWI should be sought for uncertain cases.

## 1. Introduction

Cervical artery dissection (CAD) is one of the major causes of stroke in young and middle-aged adults, often caused by neck distortion, chiropractic manipulation, and trauma (1–3). Patients with CAD can have a wide range of symptoms, varying from completely asymptomatic to a fatal stroke. Typically, CADs present symptoms such as headache or neck pain and a partial Horner's syndrome, followed by retinal or cerebral ischemia (4–6). Most strokes occur in the first few weeks following dissection (7). Early and accurate diagnosis would allow for timely intervention for a good prognosis.

As advanced neuroimaging techniques are becoming widely available, especially the use of three-dimensional, high-resolution, multi-contrast vessel wall magnetic resonance imaging (hrVWI), dissection may be more frequently identified (8, 9). However, currently, hrVWI is not the first choice in daily clinical practice for patients with neurological deficits due to its long scanning time. Instead, a brain MRI, including T1-weighted imaging (T1W), T2-weighted imaging (T2W), T2-fluid attenuated inversion recovery (T2-FLAIR), and diffusion-weighted imaging (DWI), is first performed for most of those patients as part of their standard etiologic checkup for the early detection of cerebral ischemia. Previously, CT was used as the first choice for acute ischemic stroke (AIS). In recent years, with the popularity of the MRI system and the acceleration of image acquisition, MRI has been increasingly used for AIS assessment. In our hospital, a green channel has been established, and MRI is available for patients with acute neurological symptoms.

Cervical artery dissections are often located in the extracranial internal carotid artery (ICA), and the tear frequently extends cranially to the petrous segment (10, 11). Therefore, CAD can be detected using images obtained from the standard brain MRI as it usually covers the subpetrous segment of the ICA (12, 13). With this relevance, the main aim of this study was to assess the accuracy of detecting dissection in the extracranial ICA based on the combination of a standard brain MRI and patient clinical information. The complementary value of hrVWI in CAD detection was also assessed.

## 2. Materials and methods

### 2.1. Patients

In this retrospective study, consecutive patients with CAD at the First Affiliated Hospital of Zhengzhou University, Zhengzhou, China, between January 2019 and April 2022 who underwent standard brain MRI and hrVWI were screened by reviewing imaging examinations, laboratory tests, discharge reports, and other medical records. This study was approved by the local institutional review board, and written informed consent from the patient was waived. All patients had neurological symptoms such as headache or neck pain, dizziness, motor or sensory disorders, transient ischemic attack, or ischemic stroke. MR imaging was performed within 7 days after symptom onset. A total of 184 patients were identified, of which 79 of them were excluded for various reasons, including missing data, poor image quality, and dissection not located in the cervical artery. Finally, 105 patients with 123 CADs were included in the analysis.

Another 105 patients without CAD were included as controls. The control patients were individually matched to each case patient, within corresponding gender, age, National Institutes of Health Stroke Scale(NIHSS) score, and infarct on DWI (including infarct location and size) and a scanning machine. The lesion type in this group was assessed according to all available images, including brain MRI, CTA, hrMRI, and DSA. Standard brain MRI and hrVWI were also performed in the control group.

### 2.2. MR imaging and image analysis

**Figure 1:**
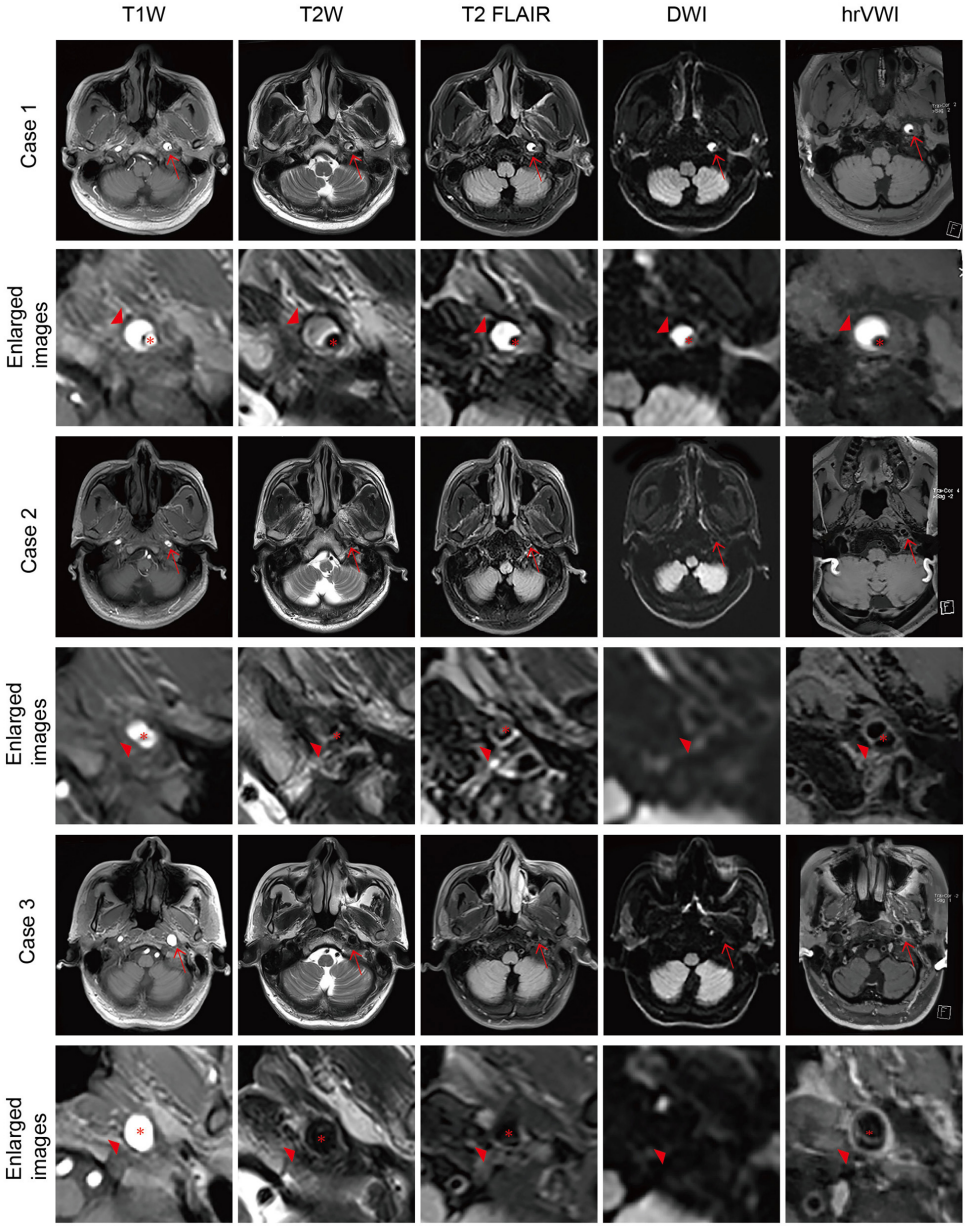
Representative brain MRI and high-resolution vessel wall magnetic resonance imaging (hrVWI) of extracranial internal carotid artery (EICA) dissection with typical signs. **(Case 1)** The dissection in the distal third of the left EICA with a mural hematoma on axial brain MRI and hrVWI sequences (arrow), showing crescentic hypersignals (arrowhead) with a tiny lumen. **(Case 2)** A left EICA dissection with an intimal tear/double lumen on axial brain MRI and hrVWI sequences (arrow), showing an isoisointense line crossing the dilated lumen (arrowhead). The lesion is ambiguous on DWI and distinct in other sequences. **(Case 3)** A left ICA dissection with a dissecting aneurysm on axial brain MRI and hrVWI sequences (arrow). The lesion is negative on DWI and shows vessel dilation on other sequences (arrowhead). The intimal flap is only seen on the hrVWI. The red asterisk indicates the lumen.

**Figure 2:**
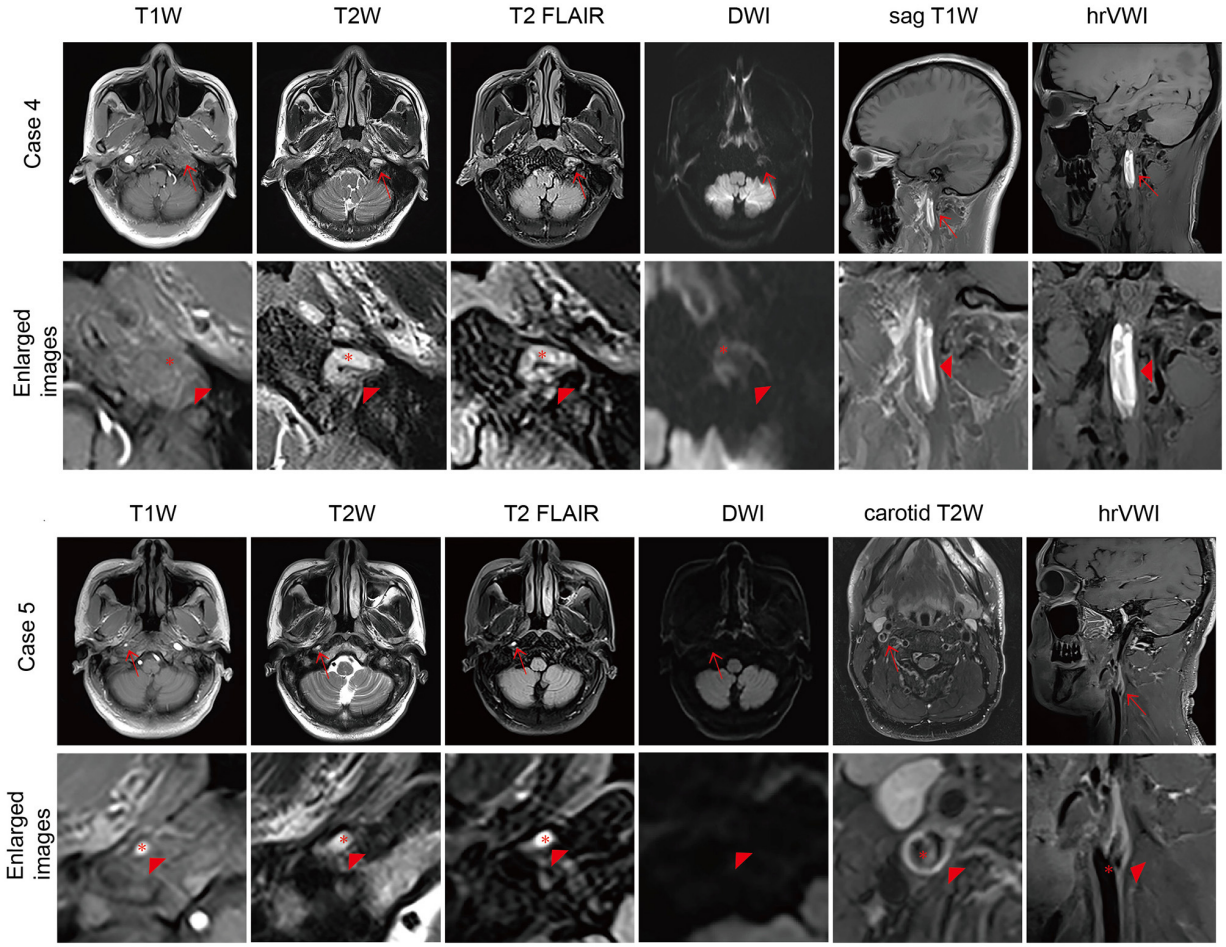
Representative brain MRI and high-resolution vessel wall magnetic resonance imaging (hrVWI) of extracranial internal carotid artery (EICA) dissection with non-specific signs. **(Case 4)** A dissection with mural hematoma in the distal two-thirds of the left EICA on brain MRI and hrVWI sequences (arrow). Axial T2W and T2 FLAIR images show kinking hypersignal on the left EICA (arrowhead), while axial T1W and DWI images are equivocal (arrowhead); sag T1W and hrVWI images show obvious stripped hypersignal (arrowhead). **(Case 5)** A dissection with an intimal flap in the right proximal EICA (arrow). Axial T1W, T2W, and T2 FLAIR images show tiny hypersignals on the right EICA while DWI is negative (arrow and arrowhead). A curvilinear line is located in the lumen of the right proximal EICA on hrVWI and carotid T2W images (arrow and arrowhead). The red asterisk indicates the lumen.

MRI examinations were obtained using a 3.0 Tesla MR scanner (Verio, Skyra, or Prisma, Siemens Healthcare, Erlangen, Germany). The brain MRI protocol included the following four axial sequences with 5-mm slice thickness: T1W using Fast Low Angle Shot (FLASH) (TR/TE: 220/2.5 ms; matrix: 320 × 256; FOV: 230 × 230 mm^2^; acquisition time: 28 s); T2W using turbo spin echo (TR/TE: 5,000/117 ms; matrix: 448 × 336; FOV: 230 × 230 mm^2^; acquisition time: 39 s); T2 FLAIR (TR/TE: 6,500/85 ms; matrix: 320 × 224; FOV: 230 × 230 mm^2^; acquisition time: 1 min 15 s); DWI using spin-echo echo-planar imaging (TR/TE: 4,600/80 ms; matrix: 192 × 192; FOV: 230 × 230 mm^2^; acquisition time: 47 s); and one sagittal T1W sequence with 5-mm slice thicknes using FLASH(TR/TE: 240/2.5ms; matrix: 320 × 224; FOV: 230 × 230 mm2; acquisition time: 30 s) . The anatomy coverage of axial sequences extended from the vertex to the upper part of the second cervical vertebra, including the distal, subpetrous cervical segment of the cervical ICA and the sagittal T1W was acquired from one side of the scalp surface to the other. In addition to 3D time-of-flight (TOF) angiography, the hrVWI was acquired using a three-dimensional turbo spin echo technique known as T1W-sampling perfection with application-optimized contrast using different flip angle evolutions (T1-SPACE) and two-dimensional axial T2W using turbo spin echo. The parameters were as follows: TR/TE: 900/15 ms; matrix: 320 × 320; FOV: 230 × 230 mm^2^; slices number: 224; slice thickness: 0.6 mm; voxel size: 0.6 × 0.6 × 0.6 mm^3^; acquisition time: 8 min and TR/TE: 4,000/62 ms; matrix: 256 × 256; FOV: 160 × 160 mm^2^; slices number: 21; slice thickness: 2 mm, respectively. Representative cases are shown in Figures 1, 2.

The image analysis was performed independently by two radiologists (SX was a radiologist with 10 years of experience, and YR was a radiologist with 8 years of experience in vessel wall imaging), and one of them (SX) repeated each type of analysis 4 weeks later. The lesions were classified into not CAD (G1), not sure (G2), and CAD (G3) based on the information available. For the diagnosis of CAD, the two radiologists first analyzed brain MR images while being blinded to all clinical information, hrVWI, and images from other modalities, including magnetic resonance angiography (MRA), digital subtraction angiography (DSA), and computed tomography angiography (CTA). Image signs in brain MRI suggesting CAD included a crescentic or circular iso- or hyperintensity around the lumen, a curvilinear and isointense line crossing the lumen, or aneurysmal vessel dilation (Figure 1). Image reading for CAD diagnosis was repeated unblinded to information of outpatient inquiries and patient clinical presentations but blinded to hrVWI and images from other modalities. During the outpatient/emergency unit inquiry, patients were asked questions to assist in the identification of the cause of CAD. Possible inducements included trauma, cervical manipulation, neck distortion, vigorous exercise, migraine, pregnancy, and recent infection. Typical patient presentations suggesting CAD included headache, neck pain, and Horner's syndrome. Further image reading in combination with brain MRI, patient clinical information, and hrVWI was performed to assess the complementary value of hrVWI for CAD diagnosis. The diagnosis at the end based on the clinical information and all available imaging data (including brain MRI, hrVWI, MRA, CTA, DSA, and ultrasonography) according to the consensus published in 2015 (14) served as the gold standard in this study (15–17).

### 2.3. Statistical analysis

Continuous data were presented as mean and standard deviation (mean ± SD) or median and interquartile range (IQR), depending on their distribution. Categorical data were expressed as counts (percentage). Student's *t*-test, Mann–Whitney *U*-test, and χ^2^ test were used to assess the difference between the two groups where appropriate. The sensitivity, specificity, positive predictive value (PPV), and negative predictive value (NPV) were calculated to quantify the accuracy of the CAD diagnosis. Cohen's *κ* coefficient was calculated to measure the intra- and inter-observer agreements. The *κ* value was interpreted as follows: >0.80, suggesting an excellent agreement; 0.61–0.80, good; 0.41–0.60, moderate; 0.21–0.40, fair; and 0–0.21, poor (18). A 2-sided *P*-value of < 0.05 was considered statistically significant. All statistical analysis was performed using SPSS 20.0 (IBM, NY, USA).

## 3. Results

In total, 105 patients with CAD and 105 patients without CAD who served as controls were enrolled in this study. There were no significant differences in patient demographics, NIHSS score, and other risk factors (Table 1). More patients with dissection suffered from at least one of the symptoms of headache, neck pain, or Horner's syndrome than the controls (41.9 vs. 12.4%; *P* < 0.001).

**Table 1:** Patient demographics.

	**CAD (*n* = 105)**	**Controls (*n* = 105)**	***t*/χ^2^/*Z***	***P*-value**
Age, year, (mean ± SD)	47.2 ± 11.0	46.6 ± 10.9	0.373	0.709
Men, *n* (%)	80 (75.2)	80 (75.2)	0.00	1.00
NIHSS, median (IQR)	1 (0, 2)	1 (0, 2)		0.468
DWI positive, *n* (%)	76 (72.4)	76 (72.4)	0.00	1.00
Onset to MRI time < 72 h, *n* (%)	24 (22.9)	32 (30.5)	1.558	0.212
Risk factors, *n* (%)	11 (10.5)	5 (4.8)	2.436	0.119
Cervical manipulation, *n* (%)	3 (2.9)	0 (0)		
Trauma, *n* (%)	2 (1.9)	0 (0)		
Vigorous exercise, *n* (%)	1 (1.0)	1 (1.0)		
Migraine, *n* (%)	4 (3.8)	4 (3.8)		
Pregnancy, *n* (%)	1 (1.0)	0 (0)		
Clinical manifestation, *n* (%)	44 (41.9)	13 (12.4)		< 0.001
Headache, *n* (%)	31 (29.5)	13 (12.4)		
Neck pain, *n* (%)	7 (6.7)	0 (0)		
Horner's syndrome, *n* (%)	6 (5.7)	0 (0)		

Of patients with CAD, 61 of them had ultrasonography, 95 had CTA, and 74 had DSA; in the control group, 71 of them had ultrasonography, 89 had CTA, and 87 had DSA. Among the 105 patients with CAD, 123 dissections were found (18 patients with bilateral dissections) based on the clinical information and all available imaging data. Of these 123 dissections, 31 (25.2%) were located in the proximal third of the cervical ICA, 4 (3.3%) in the proximal two-thirds, 5 (4.1%) in the middle third, 39 (31.7%) in the distal third, 26 (21.1%) in the distal two-thirds, and 18 (14.6%) propagated along the whole course.

### 3.1. Detection of cervical artery dissection based on brain MRI

Based on brain MRI alone, 57 patients with 62 dissections were correctly diagnosed in the dissection group, and 3 patients were misdiagnosed as having dissection (G3) in the control group. The frequencies of specific imaging signs suggesting the dissection were 48 for a crescentic or circular sign, eight for a curvilinear sign, and six for aneurysmal vessel dilation, respectively. A total of 87 ICAs in the dissection group and 207 ICAs in the control group were correctly diagnosed (G1). In the dissection group, 49 lesions were rated as G2 (not sure). The brain MRI alone offered diagnostic performance per lesion with an AUC of 86.6% (95%CI: 82.0–91.3%), high specificity (99.0%), and moderate sensitivity (50.4%) (Table 2). These imply that if a brain MRI suggests a CAD, the lesion is very likely a CAD, but its accuracy in excluding its existence is marginal. The assessment at the patient level is also listed in Table 2.

**Table 2:** Diagnostic performance of brain MRI and hrVWI with and without clinical information for the detection of dissection located in the internal carotid artery (ICA) at the patient and artery levels.

	**AUC % (95%CI)**	**Youden index**	**Sensitivity % (*n*/*N*)**	**Specificity % (*n*/*N*)**	**PPV % (*n*/*N*)**	**NPV % (*n*/*N*)**	**Inter-observer (κ)**	**Intra-observer (κ)**
Per-patient								
Brain MRI	84.7 (79.2–90.2)	0.514	54.3 (57/105)	97.1 (102/105)	95.0 (57/60)	68.0 (102/150)	0.832	0.896
Brain MRI + clinical information	87.8 (82.6–92.9)	0.724	73.3 (77/105)	99.0 (104/105)	98.7 (77/78)	78.8 (104/132)	0.855	0.890
hrVWI	99.3 (98.2–100)	0.886	95.2 (100/105)	93.3 (98/105)	93.5 (100/107)	95.1 (98/103)	0.911	0.920
hrVWI + clinical information	99.4 (98.3–100)	0.914	96.2 (101/105)	95.2 (100/105)	95.3 (101/106)	96.1 (100/104)	0.929	0.938
Per-artery								
Brain MRI	86.6 (82.0–91.3)	0.494	50.4 (62/123)	99.0 (294/297)	95.4 (62/65)	82.8 (294/355)	0.915	0.939
Brain MRI + clinical information	89.2 (84.8–93.5)	0.712	71.5 (88/123)	99.7 (296/297)	98.9 (88/89)	89.4 (296/331)	0.952	0.964
hrVWI	99.4 (98.3–100)	0.921	95.1 (117/123)	97.0 (288/297)	92.9 (117/126)	98.0 (288/294)	0.930	0.935
hrVWI + clinical information	99.8 (99.4–100)	0.936	95.9 (118/123)	97.6 (290/297)	94.4 (118/125)	98.3 (290/295)	0.941	0.946

AUC, area under the curve; CI, confidence interval; PPV, positive predictive value; NPV, negative predictive value; MRI, magnetic resonance imaging; hrVWI, three-dimensional, high-resolution, multi-contrast vessel wall MR imaging.

The inter-observer agreement for dissection detection was excellent (*κ* = 0.832, *P* < 0.001 per patient and *κ* = 0.915, *P* < 0.001 per lesion). More comparisons are shown in Table 2.

### 3.2. Detection of cervical artery dissection based on the combination of brain MRI and patient clinical information

When patient clinical information, including patient inquiry and clinical presentations, was unblinded, 23 lesions rated as G2 and three lesions rated as G1 previously were correctly classified as G3, and two arteries rated as G3 were corrected as G1. Out of 105 patients, 77 (73.3%) patients with dissection and 104 (99.0%) without dissection were correctly identified. The combination of brain MRI and patient clinical information offered diagnostic performance per lesion with an AUC of 89.2% (95% CI: 84.8–93.5%) (Table 2). Clinical information helped in improving the diagnostic accuracy for uncertain cases based on brain MRI alone. The assessment at the patient level is also listed in Table 2.

The inter-observer agreement for dissection detection was excellent at both patient (*κ* = 0.855, *P* < 0.001) and lesion levels (*κ* = 0.952, *P* < 0.001) when brain MRI and patient clinical information were combined (Table 2).

### 3.3. Detection of cervical artery dissection based on hrVWI alone and the combination of patient clinical information and hrVWI

Based on hrVWI alone, 117 CADs were diagnosed from 100/105 (95.2%) patients in the dissection group, and 94.0% (109/117) of them were correct when referred with the gold standard; the diagnosis could not be made for six lesions from five patients. The frequencies of pathognomonic signs of the internal carotid artery dissections were 59 intramural hematomas, 29 intimal flaps, two double lumens, 14 dissecting aneurysms, three intramural hematomas with intimal flap, one intramural hematoma with double lumen, and one double lumen with intimal flap. In the control group, one lesion was wrongly diagnosed as CAD, and a decision could not be made for eight lesions from six patients. Totally, 288 lesions from 190 patients were correctly diagnosed as not having CAD. Based on the combination of hrVWI and clinical information, one lesion previously rated as G2 was correctly classified as G3, and two arteries rated as G2 were corrected as G1. HrVWI alone offered diagnostic performance per lesion with an AUC of 99.4% (95% CI: 98.3–100.0%), and the sensitivity was 95.1%, specificity was 97.0%, PPV was 92.9%, and NPV was 98.0%. The hrVWI cooperating patient clinical information offered diagnostic performance per-lesion analysis with AUC values of 99.8% (95% CI: 99.4–100.0%) (Table 2). The assessment at the patient level could be found in Table 2.

The inter-/intra-observer agreements for dissection detection based on hrVWI and the combination of hrVWI and clinical information were excellent (*κ* = 0.930, *P* < 0.001 and *κ* = 0.941, *P* < 0.001, respectively) (Table 2).

## 4. Discussion

This study showed that 54.3% of dissections located in the extracranial ICA could be correctly identified by a routine brain MRI, and when the clinical information was incorporated, 73.3% of them could be detected. Typical clinical signs included headache, neck pain, and Horner's syndrome, and representative imaging signs in the brain MRI included a crescentic or circular iso- or hyperintensity around the lumen, a curvilinear and isointense line crossing the lumen, or aneurysmal vessel dilation (Figure 1). This study found that hrVWI had a superior capability in detecting CAD than brain MRI, both with and without the combination of patient clinical information.

This study suggests that, in daily clinical practice, (1) in hospitals where hrVWI cannot be performed; (1.1) neuroradiologists should remind neurologists about the possibility of CAD when typical imaging signs in the standard brain MRI are found. On the other hand, neurologists should provide the clinical information for suspected cases to neuroradiologists for a more accurate imaging report; (1.2) other imaging modalities should be sought for further investigation for patients with suspected CAD but without typical imaging signs in the brain MRI; and (2) in hospitals where hrVWI can be performed, hrVWI should be the first choice when a patient possibly suffers from a CAD. Since hrVWI is available in most commercial 1.5 Tesla and 3.0 Tesla MR scanners, the implementation of hrVWI should be encouraged. However, it is challenging to implement hrVWI sequences and interpret the images acquired. Routine training on these two aspects should be provided.

The causes of CAD and associated clinical presentations vary widely. CAD might be the result of genetic predisposition, trauma, cervical manipulation, migraine headaches, pregnancy, and sports (1–3, 19–21). In this study, 11 patients with dissection had such risk factors compared with five in the control group (*P* = 0.119). The symptoms of CAD varies from asymptomatic to severe stroke. It typically begins with an ipsilateral headache, neck pain, and a partial Horner's syndrome, followed by retinal or cerebral ischemia. The presence of any two of the three presentations strongly suggests CAD (4). After incorporating this clinical information, the accuracy of CAD diagnosis increased by 20%. For patients without CAD-specific imaging features on brain MRI but with clinical information suggesting CAD, further examinations should be performed promptly.

The value of standard brain MRI for the detection of CAD in the upper portion of carotid and vertebral arteries had been evaluated previously. Naggara et al. (12) explored the capacity of diagnosing a dissection involving the upper cervical portions of carotid arteries by analyzing five different brain sequences (sagittal T1W, axial FLAIR, axial T2W, DWI, and 3D TOF). Out of the 77 patients with CAD and 77 without CAD, 59 (76.6%) and 73 (94.8%) patients, respectively, were correctly classified. A recent study (22) reported that nearly 90% of cervical ICA dissections at the acute phase of a stroke could be identified using a standard DWI sequence. The intramural hematoma was a common and typical imaging feature in reported studies for the diagnosis of dissection. In addition to intramural hematoma (*n* = 72), intimal flap/double lumen (*n* = 39) and pseudoaneurysm (*n* = 12) are also typical imaging signs, as shown in this study. Notably, due to the degeneration of intramural hematoma, signal intensity varies over time on images with different weighting, e.g., regarding the healthy arterial wall, the hyperacute hematoma appears isointense on T1W and hyperintense on T2W images, early acute hematoma appears hyperintense on T1W and hypointense on T2W images, and late acute hematoma appears hyperintense on both weighted images (23).

The overall sensitivity in this study was lower (54.3%) in the detection of extracranial ICA dissections based on the standard brain MRI, and it increased to 73.3% when patient clinical information was combined compared with previous reports, e.g., 76.6% by Naggara et al. (12). In particular, the accuracy was as high as 93.1% for carotid dissections (54/58) (but it was low for vertebral dissection, 5/19) (12). The difference might be due to the use of 3D TOF in the study by Naggara et al. (12), which is usually not a common sequence included in the standard brain MRI. Approximately 40% of the dissections in this study were not detectable by brain MRI. These lesions were mostly located in the proximal or middle part of the ICA and did not cause lumen stenosis or thrombosis downstream. More than half of the non-detectable dissections manifest as non-specific findings, such as a reduced diameter of the ICA due to the insufficient blood flow due to proximal stenosis caused by dissection, which is particularly obvious on axial T1W as the ICA shows a high signal using gradient recalled echo. The dissected ICA could also show a normal diameter with hypersignal in the lumen due to thrombosis, especially on T2W and T2 FLAIR, as the ICA shows flow voids using the spin echo technique (Figure 2). These situations should be distinguished from atherosclerotic stenosis or thrombosis, which need clinical information and further examination. Another non-specific finding is irregular dilation, which is easily confused with ICA redundancy. While arterial tortuosity is associated with spontaneous cervicocerebral artery dissection (24, 25), dissection should be taken into consideration in such cases.

Over the past two decades, hrVWI has evolved from two-dimensional acquisition (26–28) with several slices along the artery and a small anatomy coverage (40–60 mm) to three-dimensional acquisition with a large coverage (150–200 mm) (29–31). HrVWI has been established as a powerful diagnostic tool for atherosclerosis and aneurysm detection and quantification in the head and neck (32). It is capable of directly visualizing specific CAD, including the intramural hematoma, double lumen or intimal flap, and aneurysmal dilation (8, 33), thus improving the accuracy of CAD detection (15).

Despite useful findings, limitations exist: (1) this is a single-center, retrospective study with a small sample size (105 patients with dissections and 105 patients without dissections); (2) follow-up imaging had not been planned systematically, and chronological change in morphology was therefore not analyzed in this study; (3) since no open surgery was performed for any of these patients, the true gold standard was not available, and the lesion assessment was performed according to the consensus published in 2015 in this study; (4) imaging using other modalities, e.g., DSA, and CTA, was not performed for all patients, and 3D T2-weighted sequences, such as T2-weighted SPACE, was not included in the hrVWI protocol in this study; and (5) patients with lesions located in the posterior circulation were not included.

## 5. Conclusion

This study provides evidence that brain MRI performed routinely accompanied by clinical findings can be an effective method in the early detection of extracranial ICA dissections, and hrVWI showed a superior capability in detecting ICA dissections.

## Data availability statement

The original contributions presented in the study are included in the article/supplementary material, further inquiries can be directed to the corresponding authors.

## Ethics statement

The studies involving human participants were reviewed and approved by Institutional Review Board of the First Affiliated Hospital of Zhengzhou University (Zhengzhou, China). The patients/participants provided their written informed consent to participate in this study.

## Author contributions

SX contributed to the study design, data collection and analysis, and drafting and revising the manuscript for content. YRa, XW, and QF performed data analysis, interpretation, and statistical analysis. YZ and JL took part in the study concept and design. YRe participated in data acquisition. ZT and JC contributed to the study concept and design, data and results interpretation, and revising the manuscript significantly. All authors read and approved the final manuscript.
